# Recent Progress in Understanding the Health Benefits of Curcumin

**DOI:** 10.3390/molecules28052418

**Published:** 2023-03-06

**Authors:** Chiara Porro, Maria Antonietta Panaro

**Affiliations:** 1 Department of Clinical and Experimental Medicine, University of Foggia, 71121 Foggia, Italy; 2 Department of Biosciences, Biotechnologies and Environment, University of Bari, 70125 Bari, Italy

Nutrients and their potential benefits are a new field of study in modern medicine due to their positive impact on health. Several studies have reported that a diet rich in antioxidants and anti-inflammatory compounds may decrease age-related cognitive decline and the risk of developing various diseases [1,2]. Among natural compounds, curcumin, the main active component isolated from the rhizome of *Curcuma Longa* L., is well known for its beneficial effect on human health [3]. The (1*E*,6*E*)-1,7-bis(4-hydroxy-3-methoxyphenyl)-1,6-heptadiene-3,5-dione is the IUPAC name of curcumin, its chemical formula is C_21_H_20_O_6_, and it has a molecular weight of 368.38 g/mol. Various biological activities and therapeutic properties of curcumin are due to its chemistry; in particular, phenolic hydroxyl groups, the central bis-α, β-unsaturated β-diketone, double conjugated bonds, and methoxy groups are responsible for its bio-pharmacological effects (Figure 1). Curcumin was introduced in Europe in the 14th century as a culinary spice [4]. This natural compound has been used for centuries in traditional Indian Ayurvedic and Chinese medicine for treating respiratory and liver disorders, infections, allergies, and rheumatisms [5]. *C. longa* grows in tropical and subtropical climates. India is the world’s largest producer of turmeric, which has long been used as a home cure for various diseases [6,7].

In this Special Issue (SI) of the MDPI journal *Molecules* entitled “Recent Progress in Health Benefits from Curcumin”, we report the recent advances concerning the biological properties of curcumin in vitro and in clinical trials; its bioavailability; and its health-promoting effects other than its potential therapeutic applications, including its incorporation into novel functional matrices.

Aging is known as a process in which there is an irreversible and progressive decline of physiological functions; this loss could lead to the most important age-related diseases, such as cardiovascular diseases, musculoskeletal disorders and arthritis, neurodegenerative diseases, and cancer [8]. Different aging mechanisms have been discovered, including genomic instability, telomere shortening, epigenetic changes, mitochondrial dysfunction, cellular senescence, stem cell exhaustion, and altered intercellular communication [9]. Curcumin, like other polyphenols, has pleiotropic activity. Indeed, due to its capacity to interact with many proteins, curcumin can exert cellular response to external stimuli. Moreover, curcumin up- and downregulates different miRNAs and takes part in epigenetic changes in the cell [10]. Benameur et al. evidenced that curcumin exerts anti-aging proprieties by acting on different target proteins, inducing antioxidant and anti-inflammatory processes, modulating microglia neuroprotection, and finally, acting on telomerases to arrest cancer progression [11].

Additionally, Cianciulli et al. studied the impact of curcumin on aging; they analyzed the contribution of curcumin on inflammaging. Inflammaging is a word used to describe the tight relationship between low-grade chronic inflammation and aging that occurs during physiological senescence in the absence of evident infection. This condition has been linked to a broad spectrum of age-related disorders in various organs, including the brain. Inflammaging represents a highly significant risk factor for the development and progression of age-related conditions, including neurodegenerative diseases characterized by the progressive dysfunction and degeneration of neurons in the brain and peripheral nervous system. In the brain, curcumin acting on microglia can inhibit the formation of reactive oxygen species and other pro-inflammatory mediators that are believed to play a pivotal role in many age-related diseases, such as Alzheimer’s disease (AD), Parkinson’s disease (PD), and Huntington’s disease (HD) [12].

The neuroprotective effects of curcumin were also emphasized by Benameur et al. in a review in which the authors focused their attention on the therapeutic effects of curcumin in neurodegenerative disorders such as AD, PD, multiple sclerosis, glioblastoma, and epilepsy by modulating various molecular pathways in brain cells. In this review, they also described drug delivery systems to enhance the bioavailability and blood–brain barrier (BBB) permeability of curcumin to optimize the use of curcumin in the prevention and treatment of neurodegenerative diseases [13].

Curcumin can induce neuroprotection in methamphetamine-induced toxicity in catecholamine cells PC12, confirming its potential power not only to prevent neurodegenerative diseases but also to counteract the neurotoxic effects induced by drugs [14].

The effects of curcumin were also seen on malignant brain tumors of the central nervous system (CNS).

In particular, Gallien and colleagues reported that curcumin encapsulated in surface-modified polyamidoamine dendrimers reduced the viability of three glioblastoma cell lines, mouse-GL261, rat-F98, and human-U87, compared to non-cancerous control cells, attesting its anti-tumor proprieties [15].

The anti-tumor properties of curcumin are not only circumscribed to a reduction in cell proliferation. In this regard, Ryskalin and colleagues focalized their attention on the capacity of curcumin to modulate autophagy-(ATG)-dependent molecular pathways.

Most of the antitumoral effects of curcumin rely on the mammalian target of rapamycin(mTOR)-dependent ATG induction. In fact, curcumin targets undifferentiated and highly tumorigenic GBM cancer stem cells (GSCs) are suppressed through the modulation of the mTOR-dependent ATG pathway, suppressing the tumorigenic features of GSCs that reduce GBM growth [16].

Leksiri and co-authors investigated the effect of the co-administration of pregabalin– curcumin in attenuating acute nociceptive pain without a noticeable impact on motor function. It has been demonstrated that the main action of pregabalin is modulating neurotransmitters in the spinal cord, though some studies also found its action probably to be in the dorsal root ganglia [17,18]. The main effect of curcumin as an anti-inflammatory agent and TRPV1 (transient receptor potential cation channel subfamily V member 1) antagonist is likely in the peripheral immune system due to its inadequate BBB penetration. Although some studies reported that curcumin could decrease the activated resident glia in the spinal cord [19], the action might be mainly via an indirect mechanism of reducing peripheral sensitization rather than acting directly on the CNS. Therefore, the synergistic analgesia observed with the combination of pregabalin and curcumin is due to the ability of the two drugs to act on both the peripheral and central nervous systems concurrently [20].

The combination of curcumin with another polyphenol, such as resveratrol, improved muscle function and structure in experimental cancer-induced cachexia.

Treatment with resveratrol and curcumin of the cancer cachectic mice elicited favorable effects in a similar fashion in both slow- and fast-twitch limb muscle types. Interestingly, a significant rise in sirtuin-1 protein content was detected in the gastrocnemius and soleus muscles of mice treated with either curcumin or resveratrol. These relevant findings suggest that the benefits observed in the cachectic limb muscles are mediated to a great extent by the actions of sirtuin-1. Furthermore, skeletal muscle damage, a common feature in cancer-induced cachexia [21,22], also decreased in animals treated with either of the two polyphenolic compounds compared to nontreated mice [23].

Regarding the combination of curcumin with other therapies, Kong et al. provided critical insight into the mechanisms of action of curcumin and its role in combination with other cancer therapies in different types of cancers, also focusing on the molecular target involved [24].

Different limitations of the clinical application of curcumin are represented by poor bioavailability and low solubility and stability, which are related to its hydrophobic structure. Due to this, different studies have analyzed innovative biotechnological strategies to increase the bioavailability and efficacy of curcumin.

In this regard, three reviews in this Special Issue have summarized new nanoformulations of curcumin.

Chopra et al. discussed nanoformulation techniques that enhance the characteristics of curcumin, such as increasing bioavailability and solubility, modifying metabolism and target specifity, prolonging circulation, and enhancing permeation. They report in this review new inventions for nanoformulation [25].

Nanoparticles (NPs) are drug delivery systems that can increase the bioavailability of hydrophobic drugs and improve drug targeting to cancer cells via different mechanisms and formulation techniques. In the review of Amekyeh et al., various CUR-NPs and their potential use in treating cancers are analyzed. This review evaluated different formulations, including lipid, gold, zinc oxide, magnetic, polymeric, and silica NPs, as well as micelles, dendrimers, nanogels, cyclodextrin complexes, and liposomes, with an emphasis on their formulation and characteristics. The authors reported that curcumin incorporation into the NPs enhanced its pharmaceutical and therapeutic significance with respect to solubility, absorption, bioavailability, stability, plasma half-life, targeted delivery, and anticancer effect. The authors also reported that several CUR-NPs have promising anticancer activity; however, clinical reports on them are limited, so they proposed the use of many clinical trials on CUR-NPs to ensure their effective translation into clinical applications [26].

Nanoformulation was also reviewed by Tagde and colleagues, who analyzed several curcumin nanoformulations to treat various diseases in humans. The authors also confirmed the positive impact of curcumin-loaded NPs in combination with the main therapeutic agents because this formulation induces a lower dose of the main therapeutic agent, enhancing therapeutic potential while lowering systemic toxicity. In this study, they also determined that the toxicity of nanoparticles is affected by their state of aggregation and mechanical properties, which are dependent on their production and purifying methodologies. Thus, more research is needed to develop curcumin-loaded NPs with lesser toxicity. In addition, Tagde and colleagues supported the use of more in vivo tests in various disease experimental models to provide a more definite platform for promoting nanocurcumin up to the level of clinical trials [27].

Stati et al. tested curcumin–silver nanoparticles on human pterygium keratinocytes with a new formulation compatible with eye drops; this work represents an important step in the creation of an ophthalmic product able to tackle an invasive and quite severe human disease, which is now difficult to treat, let alone eliminate. Further in vitro and in vivo studies, both in animal and human models, will be necessary to test the efficacy and tolerability of this innovative compound [28].

Curcumin also exerts an in vitro anti-parasitic effect by affecting the hydrogenosomal function of *Trichomonas vaginalis*, a flagellated protist that parasitizes the human urogenital tract and causes trichomoniasis. Curcumin also exerts an anti-inflammatory effect on the host, demonstrating the potential suitability of this compound as a treatment for trichomoniasis and its ability to improve the immunopathological effects derived from the disease [29].

Besides the anti-parasitic effect, curcumin also has antimicrobial activity; in fact, curcumin-loading membranes were able to inhibit *Pseudomonas aeruginosa* and *Streptococcus mutans* biofilm growth and activity, thus representing a promising solution for the prevention of biofilm-associated infections. Moreover, the high biocompatibility and the ability to control the oxidative stress of damaged tissue make the synthesized membranes useful as scaffolds in tissue engineering regeneration, helping to accelerate the healing process [30].

The studies presented in this Special Issue have confirmed the multiple proprieties of curcumin that are beneficial to human health. More studies must be completed to improve the bioavailability of curcumin for disease treatment, focusing on new advanced drug delivery systems.

## Figures and Tables

**Figure 1:**
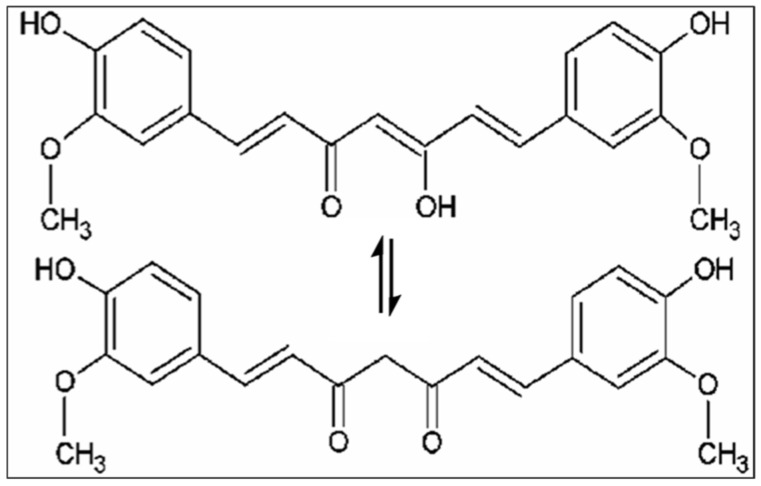
Chemical structures of curcumin.

## Data Availability

Not applicable.
